# A Simple Method for Assessing Free Brain/Free Plasma Ratios Using an *In Vitro* Model of the Blood Brain Barrier

**DOI:** 10.1371/journal.pone.0080634

**Published:** 2013-12-03

**Authors:** Maxime Culot, Anaëlle Fabulas - da Costa, Emmanuel Sevin, Erica Szorath, Stefan Martinsson, Mila Renftel, Yan Hongmei, Romeo Cecchelli, Stefan Lundquist

**Affiliations:** 1 BBB Laboratory EA 2465, IMPRT: IFR114 Université Lille Nord de France, UArtois, Lens, France; 2 DMPK department, AstraZeneca R&D, Södertälje, Sweden; Biological Research Centre of the Hungarian Academy of Sciences, Hungary

## Abstract

Historically, the focus has been to use *in vitro* BBB models to optimize rate of drug delivery to the CNS, whereas total *in vivo* brain/plasma ratios have been used for optimizing extent. However, these two parameters do not necessarily show good correlations with receptor occupancy data or other pharmacological readouts. In line with the free drug hypothesis, the use of unbound brain concentrations (Cu,br) has been shown to provide the best correlations with pharmacological data. However, typically the determination of this parameter requires microdialysis, a technique not ideally suited for screening in early drug development. Alternative, and less resource-demanding methodologies to determine Cu,br employ either equilibrium dialysis of brain homogenates or incubations of brain slices in buffer to determine fraction unbound brain (fu,br), which is subsequently multiplied by the total brain concentration to yield Cu,br. To determine Cu,br/Cu,pl ratios this way, still requires both *in vitro* and *in vivo* experiments that are quite time consuming. The main objective of this study was to explore the possibility to directly generate Cu,br/Cu,pl ratios in a single *in vitro* model of the BBB, using a co-culture of brain capillary endothelial and glial cells in an attempt to mimick the *in vivo* situation, thereby greatly simplifying existing experimental procedures. Comparison to microdialysis brain concentration profiles demonstrates the possibility to estimate brain exposure over time in the BBB model. A stronger correlation was found between *in vitro* Cu,br/Cu,pl ratios and *in vivo* Cu,br/Cu,pl obtained using fu,br from brain slice than with fu,br from brain homogenate for a set of 30 drugs. Overall, Cu,br/Cu,pl ratios were successfully predicted *in vitro* for 88% of the 92 studied compounds. This result supports the possibility to use this methodology for identifying compounds with a desirable *in vivo* response in the CNS early on in the drug discovery process.

## Introduction

The market for neuropharmaceuticals is regarded as one of the potentially largest sectors of the global pharmaceutical market owing to the increase in average life expectancy and that many neurological disorders have been largely refractory to pharmacotherapy.

The value of many promising CNS drug candidates is diminished by at the level of the cerebral capillaries of the blood-brain barrier (BBB), which possess both structural and enzymatic components. The BBB is the principal route for the entry of most molecules into the CNS as well as it is the major hurdle that prevents many neuropharmaceuticals from eliciting a desired pharmacological effect at a reasonable dose. Consequently, to have an *in vitro* model that can be used for predictions of brain uptake and investigations of therapeutic interventions at the level of the cerebral capillaries is highly advantageous. This provides not only powerful means to assess the risk for taking compounds further in the pharmaceutical development process, but also generates important information to allow for rational drug design [1].

In the late 80 and early 90’s pioneering work was performed by several groups in establishing *in vitro* models of the blood-brain barrier that could be used in an industrial setting [2]. Historically, the focus has been to use vitro models to optimize the rate of drug delivery to the CNS and *in vivo* studies to optimize extent. The rate of transport (permeability) across the BBB is a parameter that has provided CNS drug discovery research with rapid feed-back in terms of whether compounds are likely to enter the CNS in sufficient quantities or not. However, to determine a quantitative pharmacokinetic parameter that would reflect the extent of free brain exposure, researchers had to rely on *in vivo* methodologies. Knowing both rate and extent clearly contributes to a better understanding of a number of CNS indications that may require both good exposure and rapid onset of action (e.g. stroke, pain, epilepsy), whereas, for other CNS targets following chronic administration, rate may not be critical. In terms of extent, total brain/plasma ratios was for a long time the dominating parameter used for optimizing CNS exposure despite the fact that this parameter often show a poor correlation with receptor occupancy data or other pharmacodynamic readouts [3]. It was realized that only the free brain concentration (Cu,br) is available for interaction with the majority of CNS receptors and hence, of key importance. Considering the free drug hypothesis, the simplest approach would then be to assume that under freely diffusible conditions the unbound drug concentration in plasma (Cu,pl) would equal the unbound concentration in brain (Cu,br) at equilibrium. However, given the high number of compounds in CNS drug discovery programmes that are not freely diffusible across the BBB, more direct measurements were called for. Microdialysis has always been considered the “golden standard” in terms of measuring Cu,br [4] but the technique is resource-demanding and not broadly applicable because of probe recovery problems for especially, lipophilic compounds and the technique is usually reserved for compounds in later development. Cerebrospinal fluid (CSF) concentrations may provide a surrogate measure of Cu,br but are not ideal for routine screening. In addition, there may be regional variations within the CSF system depending on the time and site of sampling and also differences at the level of the blood-CSF barrier compared to the BBB, which could make CSF samples a poor indicator of Cu,br [5]. More recently, equilibrium dialysis of brain homogenates or the use of brain slices in buffer have been used to determine the free fractions of compounds in the brain (fu,br). This parameter is then used to transform *in vivo* total brain concentrations into free brain concentrations [6], [7]. However, although these are fairly straightforward techniques for determining Cu,br, both *in vitro* and *in vivo* experiments are required, which are quite time-consuming. Additional caveats with these techniques would be if the homogenate and slice methods generate significantly different results in terms of fu,br, which in turn lead to different estimations of free brain levels. Previous reports suggest for instance that variations in experimental design (homogenization, incubation times, slice thickness etc.) may influence the outcome of the experiments [8].

**Figure 1:**
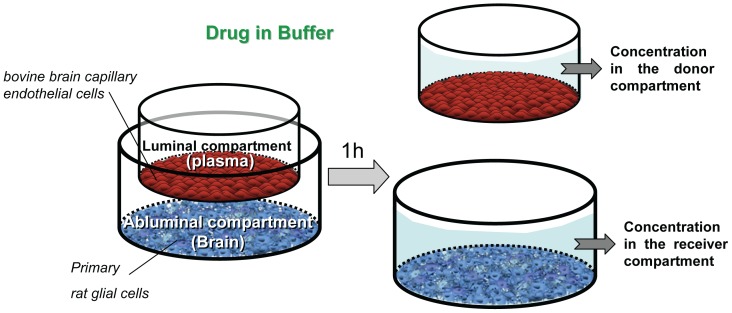
Schematic diagram of the experimental protocol. After 12 days of coculture in cell culture medium, both compartments of the *in vitro* BBB model consisting of a coculture of brain capillary endothlial cells and rat primary glial cells were filled with Hepes-buffered Ringer’s solution and the compounds dissolved at 2 µM were added in the luminal compartment (i.e donor). The presence of glial cells in the abluminal compartment was used to reproduce the non specific binding to brain tisssue within the brain prenchyma *in vivo*. After 1 hour, aliquots were taken from both compartments. *In vitro* Cu,br/Cu,pl at 1 h were calculated by dividing the concentration in the abluminal compartment (∼Cu,br) by the concentration in the luminal compartment (∼Cu,pl) at 1 h. The experimental data at 1 h were computed using the blue-norna steady-state calculator to generate the *in vitro* steady-state Cu,br/Cu,pl.

Our aim was to explore the possibility to directly generate Cu,br/Cu,pl ratios using an *in vitro* model of the BBB and thereby greatly simplifying existing experimental procedures. Since our cell culture model of the BBB consist of a co-culture of bovine brain capillary endothelial cells and rat glial cells, the presence of glial cells in the abluminal compartment was used to mimic *in vitro* the non specific binding to brain tisssue within the brain parenchyma (figure 1).

One hour after the addition of compounds dissolved at 2 µM in the luminal (i.e upper) compartment, aliquots were taken from both compartments previously filled with Hepes-buffered Ringer’s solution at the beginning of the experiment. The ratio of the compound concentration in the abluminal (i.e lower) compartment divided by the concentration in the luminal compartment was used to generate *in vitro* Cu,br/Cu,pl at 1 h. The concentrations in both compartments of the *in vitro* assay after 1 h were computed using a different calculation method accessible at blue-norna.com to generate the *in vitro* steady-state Cu,br/Cu,pl.

The main objectives of this study were to (i) compare the *in vitro* Cu,br/Cu,pl ratios generated using the BBB model with *in vivo* brain concentration profiles from microdialysis experiments. (ii) compare *in vitro* Cu,br/Cu,pl ratios *in vitro* with *in vivo* Cu,br/Cu,pl obtained using brain homogenate and brain slice methodology for a set of 32 compounds and (iii) to explore the relationship between *in vitro* Cu,br/Cu,pl and *in vivo* Cu,br/Cu,pl obtained using brain slice for a larger set of 92 compounds. Finally, the pharmacological response elicited *in vivo* for AZ13032000, a proprietary drug designed to antagonize a neuronal extracellular receptor, illustrate the possibility to use *in vitro* determined Cubr/Cupl ratios in combination with free plasma concentration profiles to establish relationships between free brain concentrations and *in vitro* efficacy data.

## Materials and Methods

### Ethics

The protocols presented below are based on animal experiments approved by the Animal Ethics Committee of AstraZeneca and University of Lille Nord de France (CEEA 382012).

### Materials

The marketed CNS compounds were obtained from Sigma-Aldrich (Stockholm, Sweden) with the exception of risperidone, olanzapine, saquinavir, donepezil, venlafaxine and ziprasidone which were obtained from Sequoia Research Products (Pangbourne, UK). [U-^14^C] sucrose (615 mCi/mmol) was obtained from Amersham (Uppsala, Sweden). All other compounds were available from in-house sources. Reagents were obtained from Sigma-Aldrich (Stockholm, Sweden).

### Plasma Protein Binding

Rat plasma was obtained from Sprague-Dawley rats in-house. Plasma from three individuals was pooled and mixed with up to 4 compounds with a concentration of 10 µM each (in 0.1% DMSO). A 48-well dialysis apparatus (AstraZeneca, Mölndal) was used to determine the plasma free fraction. The dialysis membrane (AstraZeneca, Mölndal) was conditioned in Milli/Q water before use. A volume of 180 µl 0.122 M phosphate buffer, pH 7.4 with 75 mM NaCl constituted the receiver solution and 180 µl of the plasma/compound mixture were added to the donor side. The samples were incubated on an orbital shaker (4 mm in diameter and 100 rpm) at 37oC for 18 hrs. Following incubation, 50 µl aliquots were removed from the respective plasma and buffer sides from triplicate equilibrium dialysis chambers and transferred to a 96-well assay block (Costar Brand 96-well assay blocks, polypropylene (sterile, 0.5 ml, V-bottom) (Fisher Cat No. 07200723)). After plasma protein precipitation by the addition of 150 µl of acetonitrile to each plasma sample in order to precipitate plasma proteins. The plates were centrifuged and the supernatant analyzed by LC-MS/MS. The fu in plasma was calculated from the ratio of the MS-area of the compound in the buffer to the MS-area of the compound in the plasma.

### Assessment of Fraction Unbound Brain (fubr) - Brain Homogenate and Equilibrium Dialysis

The degree of brain binding was assessed by equilibrium dialysis. Brain homogenate (10%) was spiked with test compound at 1 µM in triplicate. In the 96-well Teflon Dialysis unit (Teflon Dialysis unit-HTD 96a (Cat. No. 1001, HTDialysis)), 125 µl of samples spiked in brain homogenate was added to one side of the dialysis chamber and phosphate buffer to the other side. The plate was then placed in a shaker/incubator to equilibrate for 18 hrs at 37°C. Following incubation, 50 µl aliquots were removed from the respective brain homogenate and buffer sides from triplicate equilibrium dialysis chambers and transferred to a 96-well assay block (Costar Brand 96-well assay blocks, polypropylene (sterile, 0.5 ml, V-bottom) (Fisher Cat No. 07200723)). After the addition of 50 µl of buffer to the brain homogenate samples, 150 µl of acetonitrile was added to each sample. Finally, 50 µl of water was added to all buffer and brain homogenate samples. The plates were centrifuged and the supernatant analyzed by LC-MS/MS.

### Assessment of Fraction Unbound Brain (fubr) - Brain Slice Method

Rats (male Sprague-Dawley) were decapitated under isoflurane anesthesia and the brains were removed carefully and immersed in ice-cold oxygenated physiological buffer at pH7.4. A 6 mm coronal section was cut with a razor and mounted with cyanoacrylate glue onto the tray of the microslicer (DTK-Zero 1 Microslicer, Dosak) after which six to eight 300 µm coronal slices from striatal areas were prepared. The slices were preincubated in 10 mL of extracellular buffer (ECF) (122 mM NaCl, 25 mM NaHCO_3_, 10 mM D-glucose, 3 mM KCl, 1.4 mM CaCl_2_, 1.2 mM MgSO_4_, 0.4 mM K_2_HPO_4_ and 10 mM HEPES) for 5 min at 37°C. The compounds were dissolved at 1 µM in the buffer and the solution was incubated with the slice for 5 h. The incubations were continuously supplied with a mixture of 5% carbon dioxide and 95% oxygen.

After 5 h, the brain slices were weighed and homogenized in 9 volumes (w/v) of ECF buffer with a sonicator (SONICS VCX 500 and VCX 130). The slice homogenates in ECF buffer were stored at −20°C prior to analysis. The concentrations of the test compound in the brain slice homogenate and in the ECF buffer was determined by LC-MS/MS.

The unbound volume of distribution Vu,brain, or free fraction fu,brain was calculated according to the following equations:
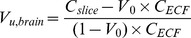


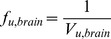
where C*_slice_*, C*_ECF_* and V*_0_* are amount of drug in the slice, the drug concentration in the ECF (representing the drug concentration in the brain ECF, i.e. the free concentration), and the water adhesion of the brain slice. V*_0_* has been estimated using ^14^C -inulin in a separate experiment in which brain slices were incubated for 1, 2, and 4 min. V*_0_* was obtained from brain slice uptake – inulin time profile (apparent zero-time intercept). V*_0_* is 0.106, i.e. on average 10.6% water is assumed to adhere to the brain slices.



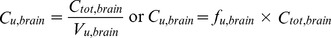
where *C_tot,brain_* is the total concentration of the compound in brain *in vivo*.

### *In vivo* Studies

#### *In vivo* brain exposure

The CNS exposure of 92 compounds (i.e 30 drugs and 62 proprietary compounds) was assessed under steady-state like conditions after intravenous administration. The time to approach steady-state (taken as at least 3.5 half-lives) was determined from prior studies of the pharmacokinetics of individual compounds after intravenous administration. The rats were put in restraint cages and cannulated via the tail vein. On the day of the experiment, the rats received a bolus injection followed by infusion. The infusion rate and infusion time were determined separately for each compound. The test formulation was administered using an infusion pump (CMA/100 or Univentor 400 microinjection pump).

Blood samples (400 µL) were collected by rapidly anaesthetizing the rats and keeping them under anesthesia with isofluran. Blood was collected either through heart puncture, from the tail vein or the orbital venous plexus and put into EDTA tubes, which were immediately placed on ice and centrifuged within 30 min at 4°C for 5 min at 2000 g to obtain plasma. The plasma was transferred by glass or plastic pipettes to 1.5 ml MTP system topaz vials and immediately frozen to −70°C or lower and stored at −20°C until assayed.

After blood collection, the whole brain was rapidly removed from the animal skull and weighed after which it was frozen and stored at −20°C. Before analysis with LC-MS/MS, the brain samples were sonicated (SONICS Vibracell VCX 130) in Ringer solution (2-fold the brain weight; e.g. 1 gram brain plus 2 mL Ringer).

The unbound brain exposure was assessed as Cu,br/Cu,pl, which is the free brain-free plasma concentration ratio. The Cu,br/Cu,pl ratios were obtained from total brain plasma ratios (Ctot,br/Ctot,pl) by using *in vitro* determined fu,br and fu,pl values according to:
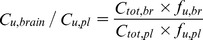



Brain concentrations were corrected for residual blood volume using 13 µl/g based on the vascular space of the marker [14C]-sucrose.

#### Brain microdialysis

The experiments were designed to evaluate the concentration-time profiles after systemic administration of sulpiride, risperidone, clozapine and 3 proprietary drugs AZ 13119277, 13032000 and 12806957, in plasma and brain extracellular fluid in freely moving rats. The commercial compounds were selected to represent different properties: Risperidone (interaction with Pgp); Clozapine (lipophilic with high permeability and high brain tissue binding) and Sulpiride (polar with low permeability and low brain tissue binding).

Adult male Wistar rats (280–350 g; Harlan, Horst, The Netherlands) were used for the experiments.

Rats were anesthetized using isoflurane 2% and O_2_. Before the surgery, Fynadine (1 mg/kg s.c.) was administered for analgesia during surgery and recovery. A mixture of Marcaine and adrenaline was used for local anesthesia on the head. Implantation of a microdialysis probe into the brain and a catheter into the right jugular vein was performed in the same surgical procedure. After surgery animals were housed individually (cages 30×30×40 cm), food and water was *ad libitum* available. Surgery was carried out at least 24 hours before the start of the experiments.

Each animal was placed into a stereotaxic frame (Kopf instruments, USA). A non-metal guide cannula with a MetaQuant probe (MQ 6/4, regenerated cellulose membrane, 4 mm exposed surface, Brainlink, The Netherlands) was implanted with in the prefrontal cortex (PFC), its tip at the following coordinates: AP = 3.4 mm (to bregma), lateral 0.8 mm (to midline), ventral −5.0 mm (to dura) with Toothbar set at −3.3 mm, according to the atlas of Paxinos and Watson (1982). The guide cannula was fixed to the skull with dental cement and a stainless steel screw.

In the same surgery procedure as described above a catheter was placed into the jugular vein to accommodate the collection of blood samples. A 10 cm segment of silicone tubing (0.64 mm ID; 0.94 mm OD) was inserted into the right jugular vein. The catheter was exteriorized through an incision on top of the head, where it was fitted onto a metal elbow. The catheter was kept patent by filling it with a 40% polyvinylpyrrolidone (40 kDa) solution containing 500 IE/ml heparin. The end of the venous catheter was fixed in position with dental acrylic cement and anchored to the skull with two stainless steel screws.

The compounds were administered at least 24 hours after surgery according to the information in table 1.

**Table 1:** Administration parameters used for brain microdialysis experiments.

Compound	Administration	Solution
Clozapine	30 µmol/kg, s.c.	10% dimethylacetamide and 20% dihydroxypropyl-beta-cyclodextrin in 0.3 M gluconic acid
Risperidone	3 mg/kg, s.c.	phosphate buffered saline with 10% glucofurol
Sulpiride	25 mg/kg, s.c.	phosphate buffered saline with 10% glucofurol
AZ12806957	30 µmol/kg, s.c.	10% dimethylacetamide and 20% dihydroxypropyl-beta-cyclodextrin in 0.3 M gluconic acid
AZ13032000	100 µmol/kg, p.o.	0.3 M gluconic acid
AZ13119277	15 µmol/kg, p.o.	10% dimethylacetamide and 30% dihydroxypropyl-beta-cyclodextrin in 0.3 M gluconic acid

On the day of the experiment, the probes were connected with flexible PEEK tubing to a microperfusion pump (Syringe pump UV 8301501, Univentor, Malta) and perfused with artificial CSF (perfusate), containing 147 mM NaCl, 3.0 mM KCl, 1.2 mM CaCl_2_, and 1.2 mM MgCl_2_ for a period of 1 hr (prestabilisation). The flow rate was 0.15 µl/min with a carrier flow of 0.80 µl/min ultrapure water. To the perfusate 0.2% bovine serum albumin (BSA) was added. Microdialysis samples were collected off-line at 30 min intervals into mini-vials by an automated fraction collector (CMA 142, Sweden), and stored at −80°C pending analysis. To verify proper flow of the slow microdialysate, all samples were collected into tared vials and weighed again. At the end of the experiment the slow flow was turned off and two more samples were collected and weighed.

Serial blood samples were collected via the jugular vein by an automated sampler (AccuSampler, Dilab, Sweden). Coagulation was prevented by adding 5 µl/vial of heparin (500 IE/ml) to each blood sample. The blood samples were centrifuged at 14,000 rpm, and plasma was stored in 1.5 ml Eppendorf vials at –80°C. After completion of the experiment, the rats were sacrificed and the brains were removed and cured in paraformaldehyde solution (4% w/v). Proper placement of each probe was verified histologically according to Paxinos and Watson (1982), by making coronal sections of the brain.

To test whether probe recoveries were sufficiently high to enable quantitative measurements of brain extracellular concentrations in the *in vivo* microdialysis experiments, each of the compounds was subjected to an *in vitro* recovery test. Microdialysis conditions were identical to those described for *in vivo* experiments, except that the probes were now placed in a standard solution of 10 nM of the respective compound, dissolved in Ringer. The bathing solution was stirred and kept at a temperature of 37°C. Each compound was tested with four probes, and from each probe a series of six samples were collected at 30 min intervals. For proper comparison, an aliquot of the bathing solution was also included with the microdialysis samples. All probe recoveries were within the acceptance limits of recovery.

Analysis of these compounds was done by LC - MS/MS detection.

All data are presented as mean values. The number of repetitions in each compound group was four. Microdialysis concentrations were corrected for dilution by the carrier flow (150 µl/min dialysate + 800 µl/min carrier solution).

### *In vitro* BBB Co-culture Model

The method of Dehouck et al. [9] was used with minor modifications (Fig. 1). Bovine brain endothelial cells isolated from capillary fragments were cocultured with primary mixed glial cells from newborn Sprague–Dawley rats. The glial cells were isolated according to the method of Booher and Sensenbrenner [10] and cultured for 3 weeks, plated on the bottom of cell culture clusters containing six wells each. The endothelial cells were seeded onto collagen-coated polycarbonate cell culture inserts (4.7 cm^2^) (Corning Incorp. NY, USA), which were placed in the wells containing glial cells. The medium used for the coculture was Dulbecco’s modified Eagle’s medium supplemented with 10% calf serum, 10% horse serum, 2 mmol/L glutamine, 50 mg/L gentamicin and 1 µg/L basic fibroblast growth factor (Sigma). The medium was changed every second day. Experiments were usually initiated after 12 days of co-culture.

Compounds were dissolved at a concentration of 2 µM in HEPES - buffered Ringer’s solution (NaCl 150 mM, KCl 5.2 mM, CaCl_2_ 2.2 mM, MgCl_2_ 0.2 mM, NaHCO_3_ 6 mM, Glucose 2.8 mM, HEPES 5 mM, water for injection) with 0.25% DMSO. To monitor the integrity of the endothelial cell monolayer, the marker molecule [^14^C] sucrose was added to the solution at a concentration of 0.05 µCi/ml. At the start of the transport experiments, buffered Ringer’s solution (pH = 7.4) was added to wells with glial cells. Inserts with brain endothelial cells were placed in these wells and drug solutions at 2 µM were added to the donor compartment. The plates were incubated for 1 h in a polymix shaker at 37°C with a low shaking velocity. Aliquots were taken from the donor compartment in the beginning and at the end of the experiments. The receiver compartments were sampled at the end of the experiments.

The amount of radiotracer in the abluminal compartment was measured in a liquid scintillation analyser (Packard Instrument Company, Meriden, USA). The BBB permeability coefficients to sucrose were calculated as described by Cecchelli et al. 1999 [11] and only results which indicated that the integrity of the monolayers was maintained for the duration of the transport experiment with drug (i.e Pe to Sucrose <0.5 10-3 cm/min) were used. The concentrations of the test compound in all samples were analysed by MS/MS.

The mean free brain/plasma ratios were calculated from three individual experiments. One hour ratios were calculated by dividing the drug concentration in the receiver compartment by the free drug concentration in the donor compartment after 1 h. The experimental data at one hour (i.e concentration in both compartment at the beginning of the experiment and after 1 h) were computed using the blue-norna® steady-state calculator (www.blue-norna.com) to predict the *in vivo* steady-state Cu,br/Cu,pl ratios. For further details on this calculation method see file S1.

### Analytical Procedures

Samples from plasma protein binding experiments were analysed using a Micromass Quattro Micro triple quadrupole (Micromass, Manchester, UK) coupled to two Shimadzu LC-10AD pumps working together as a binary pump (Shimadzu Corporation, Kyoto, Japan). A HyPurity C-18, (3 µm, 2.1×30 mm, Thermo Electron Corporation, Waltham, USA) analytical column was used. Chromatography was performed using a generic gradient at a flow rate of 0.4 ml/min. The mobile phases consisted of A: 2∶98 acetonitrile: 0.1% acetic acid (v/v) and B: 80∶20 acetonitrile:0.1% acetic acid (v/v). The gradient conditions were as follows: 0–0.2 min 0% B, 0.2–1.0 min 0–100% B, 1.0–2.5 min 100% B. The total time between injections was 3 min. The mobile phase was eluted to waste for the first 1.3 min, while data collection occurred between 1.3 and 2.5 min.

Microdialysis samples were injected onto the LC column by an automated sample injector (SIL10-ADvp, Shimadzu, Japan). Chromatographic separation was performed on a reversed-phase 150×4.6 mm Zorbax Eclipse C8, analytical column held at 30°C. The mobile phases consisted of A; ultrapure water (UP) with 0.1% Formic Acid (FA) and B; Acetonitrile with 0.1% FA. Elution of the compound proceeded using the following linear gradients at a flow rate of 0.8 ml/min.

All other samples were analysed using the following instruments: Mass spectrometer, Quattro Premier XE (Waters); autosampler, Acquity sample manager; UPLC pump, Acquity Binary solvent manager (Waters); robot for sample preparation, Biomek FX (Beckman - Coulter). The following chemicals and reagents were used: Ammonium acetate (Merck), acetonitrile gradient grade (Merck), Methanol gradient grade (Merck), Laboratory deionised water, further purified with a Milli-Q water purifying system and ammonium acetate 1 mol/L in Milli-Q water.Samples were stored in a freezer (−20°C). Prior to analysis, samples were thawed and shaken. If reanalysis was necessary, samples were stored over night at 10°C (in an auto sampler).

For chromatography the following system was used: Analytical column, Acquity UPLC BEH C18 1.7 µm 2.1×30 mm (Waters); Mobile phase A, 2% acetonitrile, 10 mM ammonium acetate and B, 80% acetonitrile in 10 mM ammonium acetate; Gradient, 2% B for 0.2 min, 2–100% B in 0.3 min, held at 100% B for 0.2 min and returned to initial condition in one step; Solvent delay 0.4 min, time between injections 1.5 min; Flow rate 0.6 ml/min; Loop: 10 µL Injection volume: 5–10 µL.

The quantification of unknown samples was performed, using QuanLynx software. Response factors were constructed by plotting peak area of the analyte against concentration of each analyte using an average response factor of the donor (D0/C0) sample injections. The average RF function without weighting was used.

## Results

The possibility to generate *in vitro* Cu,br/Cu,pl ratios at 1 hour and to predict Cu,br/Cu,pl ratio under steady-state conditions in one single experiment in a BBB model represent a great simplification over existing experimental procedures. To investigate the relationship between these two *in vitro* ratios and the *in vivo* situation. microdialysis data were collected for 6 compounds, representing three CNS drugs and three proprietary compounds. The free plasma concentrations collected in microdialysis experiments were multplied with the ratios obtained by the *in vitro* BBB model to predict *in vivo* free brain concentration-time profiles and compared with the actual free brain concentration profiles in the microdialysis experiments.

**Figure 2:**
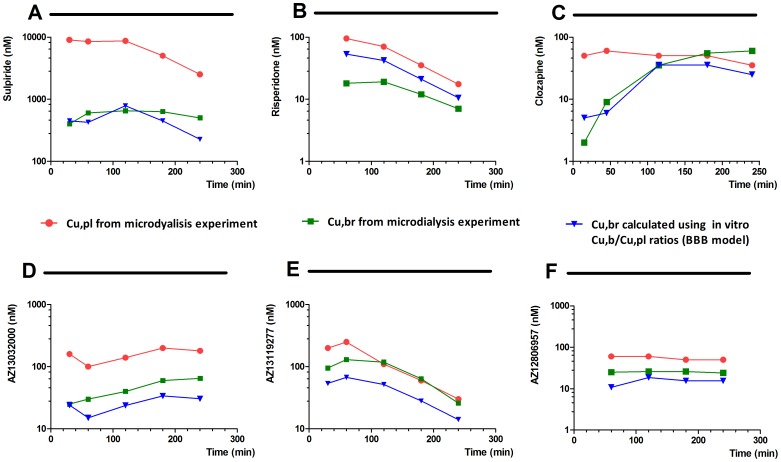
Free brain and plasma concentrations - time profile of three commercial compounds. Sulpiride (A), Risperidone (B), Clozapine (C) and 3 proprietary compounds (D, E and F) determined by brain microdialysis and compared with free brain concentrations obtained by multiplying the free plasma concentration from the microdialysis experiment with Cu,b/Cu,p ratios obtained *in vitro* using the *in vitro* BBB model. Free plasma concentration at earlier time point than 1 hour were used together with *in vitro* Cu,br/Cu,pl ratio at 1 h and microdialysis plasma concentration data at 2 hours and beyond were multiplied by the *in vitro* steady state ratio to simulate the microdialysis profile. Values for drugs are defined in Table 2 and values for proprietary compounds in Table 3.

The *in vitro* Cu,br/Cu,pl ratio at 1 h was used together with the free plasma concentration in the microdialysis experiment at earlier time point than 1 hour in order to see if it could be used to approximate the microdialysis profile up to 1 hour. By assuming that microdialysis plasma concentration data at 2 hours and beyond were representing steady state conditions, these free plasma concentrations were multiplied by the *in vitro* steady state ratio to simulate the microdialysis profile under these conditions (figure 2).

**Table 2:** Brain unbound fractions obtained using either the brain slice or the brain homogenate method and *in vitro* and *in vivo* unbound brain/plasma ratios of 30 drugs.

		fu_brain_ (homog.)		fu_brain_ (slice)		*In vitro* Cu,br/Cu,pl		*In vivo* Cu,br/Cu,pl (homog.)	*In vivo* Cu,br/Cu,pl (slice)
Compound name	Class	Mean	SD	Mean	SD	Mean 1 h	Meansteady-state	Meansteady-state	Meansteady-state
Amitriptyline	Base	0.0090	0.0005	0.0060	0.0003	0.07	0.59	1.10	0.73
Baclofen	Acid	0.5000	0.1000	0.5800	0.1610	0.05	0.06	0.02	0.02
Buproprion	Base	0.1710	0.0440	0.1600	0.0331	0.24	0.51	1.07	1.00
Caffeine	Neutral	0.5000	0.1180	0.7100	0.0780	0.34	0.55	0.48	0.68
Carbamazepine	Neutral	0.1160	0.0250	0.2000	0.0650	0.25	0.31	0.27	0.47
Carisoprodol	Neutral	0.2020	0.1200	0.5600	0.1600	0.31	0.61	0.34	0.95
Cetirizine	Base	0.0720	0.0210	0.1200	0.0510	0.03	0.01	0.01	0.01
Chlorpromazine	Base	0.0008	0.0001	0.0032	0.0005	0.06	0.69	0.12	0.49
Clozapine	Base	0.0094	0.0003	0.0100	0.0004	0.10	0.71	1.03	1.10
Diazepam	Neutral	0.0500	0.0120	0.0500	0.0215	0.16	0.32	0.49	0.49
Diphenhydramine	Base	0.0580	0.0170	0.0440	0.0130	0.13	0.55	1.38	1.05
Fexofenadine	Base	0.0770	0.0060	0.0048	0.0020	0.04	0.11	0.59	0.04
Fluoxetine	Base	0.0023	0.0001	0.0027	0.0002	0.10	0.96	0.85	1.00
Gabapentin	Base	0.7820	0.1400	0.2400	0.0430	0.06	0.14	0.46	0.14
Haloperidol	Base	0.0071	0.0003	0.0140	0.0009	0.09	0.70	0.56	1.10
Hydroxyzine	Base	0.0100	0.0060	0.0170	0.0093	0.05	0.47	0.51	0.86
Indinavir	Base	0.1000	0.0380	0.1900	0.0480	0.04	0.10	0.07	0.14
Lamotrigine	Neutral	0.2000	0.0270	0.3600	0.0350	0.27	0.43	0.34	0.61
Morphine	Base	0.5000	0.0410	0.3700	0.0370	0.14	0.31	0.20	0.15
Nortriptyline	Base	0.0046	0.0004	0.0045	0.0007	0.08	0.81	1.02	1.00
Paroxetin	Base	0.0039	0.0003	0.0024	0.0004	0.08	0.79	0.86	0.53
Phenytoin	Acid	0.0800	0.0130	0.1200	0.0070	0.22	0.44	0.28	0.42
Risperidone	Base	0.0700	0.0190	0.0700	0.0207	0.56	0.60	0.27	0.27
Sertraline	Base	0.0007	0.0004	0.0023	0.0003	0.04	0.76	0.29	1.00
Sulpiride	Base	0.6000	0.1700	0.4400	0.0270	0.05	0.09	0.07	0.05
Sumatriptan	Base	0.4000	0.0960	0.6100	0.1510	0.05	0.05	0.05	0.07
Tacrine	Base	0.1200	0.0210	0.1200	0.0330	0.23	0.58	0.78	0.78
Thioridazine	Base	0.0010	0.0002	0.0017	0.0001	0.08	0.68	0.26	0.45
Trifluoperazine	Base	0.0007	0.0001	0.0007	0.0001	0.09	0.71	1.00	1.00
Zolpidem	Base	0.2000	0.0340	0.3500	0.0456	0.25	0.49	0.24	0.42

*In vivo* Cu,br/Cu,pl were obtained from total brain plasma ratios (Cbr/Cpl) by using *in vitro* fu,br from either brain slice or brain homogenate and fu,pl according to: *Cubr/Cupl = Cbr/Cpl×fubr/fupl*. *In vivo* values were considered to approach steady-state when the time when samples were taken after at least 3.5 half-lives.

**Table 3:** *In vivo* and *in vitro* Cu,br/Cu,pl of 62 proprietary compounds.

	*In vitro* Cu,br/Cu,pl		*In vivo* Cu,br/Cu,pl (slice)		*In vitro* Cu,br/Cu,pl		*In vivo* Cu,br/Cu,pl (slice)
Compound	Mean 1 h	Meansteady-state	Meansteady-state	Compound	Mean 1 h	Meansteady-state	Meansteady-state
AZ10000568	0.03	0.14	0.03	AZ13234637	0.04	0.43	0.80
AZ10003646	0.05	0.18	0.11	AZ13242018	0.09	0.55	0.50
AZ10120857	0.06	0.74	1.00	AZ13242053	0.04	0.52	0.66
AZ10129833	0.11	0.30	0.33	AZ13246373	0.12	0.42	0.29
AZ10147710	0.04	0.03	0.03	AZ13246373	0.11	0.23	0.19
AZ10281675	0.09	0.71	1.00	AZ13250995	0.04	0.56	0.59
AZ10477650	0.07	0.04	0.01	AZ13252924	0.12	0.45	0.93
AZ10582441	0.21	0.39	0.19	AZ13258888	0.24	0.48	0.55
AZ10901745	0.22	0.46	0.80	AZ13263076	0.01	0.10	0.05
AZ11125399	0.31	0.32	0.35	AZ13294800	0.04	0.42	1.00
AZ11550918	0.19	0.24	0.34	AZ13308185	0.04	0.13	0.14
AZ11931128	0.03	0.14	0.06	AZ13312621	0.13	0.15	0.20
AZ12048147	0.05	0.11	0.09	AZ13314783	0.04	0.09	0.06
AZ12065031	0.02	0.07	0.01	AZ13317127	0.29	0.57	1.00
AZ12136053	0.12	0.04	0.12	AZ13320646	0.05	0.47	1.00
AZ12558486	0.19	0.32	0.33	AZ13328768	0.02	0.03	0.06
AZ12646603	0.19	0.45	0.29	AZ13335004	0.05	0.21	0.13
AZ12659876	0.16	0.16	0.20	AZ13342097	0.26	0.29	1.00
AZ12714134	0.20	0.69	0.40	AZ13366577	0.08	0.23	0.15
AZ12806957	0.18	0.31	0.52	AZ13366866	0.24	0.67	0.62
AZ12810256	0.14	0.31	0.30	AZ13382473	0.08	0.11	0.20
AZ12917442	0.15	0.39	0.30	AZ13384126	0.08	0.66	1.00
AZ12934216	0.19	0.30	0.22	AZ13395636	0.08	0.48	0.30
AZ12995239	0.19	0.24	0.70	AZ13403802	0.13	0.10	0.20
AZ13032000	0.17	0.15	0.19	AZ13410401	0.12	0.53	0.40
AZ13121375	0.17	0.22	0.30	AZ13411057	0.15	0.11	0.16
AZ13153303	0.11	0.49	0.60	AZ13415061	0.08	0.44	0.50
AZ13189600	0.08	0.66	0.36	AZ13428599	0.05	0.08	0.14
AZ13211419	0.05	0.54	0.85	AZ13445894	0.49	0.58	0.90
AZ13219970	0.09	0.36	0.60	AZ13447772	0.27	0.69	1.00
AZ13232626	0.10	0.52	0.40	AZ13451502	0.38	0.67	1.00

*In vivo* Cu,br/Cu,pl were obtained from total brain plasma ratios (Cbr/Cpl) by using *in vitro* determined fu,br from brain slice and fu,pl values according to: *Cubr/Cupl = Cbr/Cpl×fubr/fupl*. *In vivo* values were considered to approach steady-state when the time when samples were taken after at least 3.5 half-lives.

The commercial compounds were selected to represent different properties: Risperidone (interaction with Pgp), Clozapine (lipophilic with high tissue binding), Sulpiride (polar with low tissue binding).

All Cu,br predicted using the *in vitro* BBB model were within 3-fold of the corresponding microdialysis data except for risperidone at 30 min. These results suggest the possibility to use *in vitro* determined Cubr/Cupl ratios in combination with free plasma concentration profiles to put free brain concentrations in relation with *in vitro* efficacy data for selecting candidate drugs within the discovery phase.

The possibility of generating both non-steady state (i.e 1 h) and steady-state estimations of Cu,br/Cu,pl in the BBB model as opposed to a single ratio is a great advantage as it enable to predict brain exposure over time.

Some compounds such as sulpiride and risperidone (Fig. 2A and 2B) rapidly equilibrate between brain and blood which seems to be reflected by their close *in vitro* Cu,br/Cu,pl at 1 h and at steady-state (respectively 0.05 and 0.09 for sulpiride; 0.56 and 0.60 for risperidone). For some compounds such as clozapine (Fig. 2C), a lag in the brain concentration profile is observed relative to the plasma concentration profile in microdyalis resulting in its Cu,br/Cu,pl ranging from 0.04 at 30 min to 1.71 at 240 min. The slow equilibration of clozapine between blood and brain is reflected *in vitro* by its Cu,br/Cu,pl ratios of 0.103 at 1 h and 0.713 at steady state. In addition, as would be expected, the graphs also show that for polar and P-gp interacting compounds, both ratios predict a concentration gradient between the central and systemic compartments over time i.e. the free brain concentrations will never equal the free plasma levels, which is confirmed by the microdialysis data.

The validation of brain homogenate binding [12] and brain slice methods [7], [13] to measure unbound brain concentrations have contributed to the acceptance of Cu,br/Cu,pl as a major parameter pharmacokinetic in drug discovery. Therefore the steady-state Cu,br/Cu,pl generated using the *in vitro* BBB model were compared to the in Cu,br/Cu,pl obtained by combining *in vivo* total brain/plasma concentrations at steady-state together with the unbound fraction determined by either brain slices or brain homogenates method for a set of 30 compounds. These were selected to include both well-known CNS drugs as well as proprietary compounds covering a wide range of physicochemical properties as well as therapeutic targets. The extent of CNS exposure varies across this data set from compounds with relatively poor brain uptake *in vivo* (Cu,br/Cu,pl ratios <0.2) to those showing good CNS distribution (>0.5).

**Figure 3:**
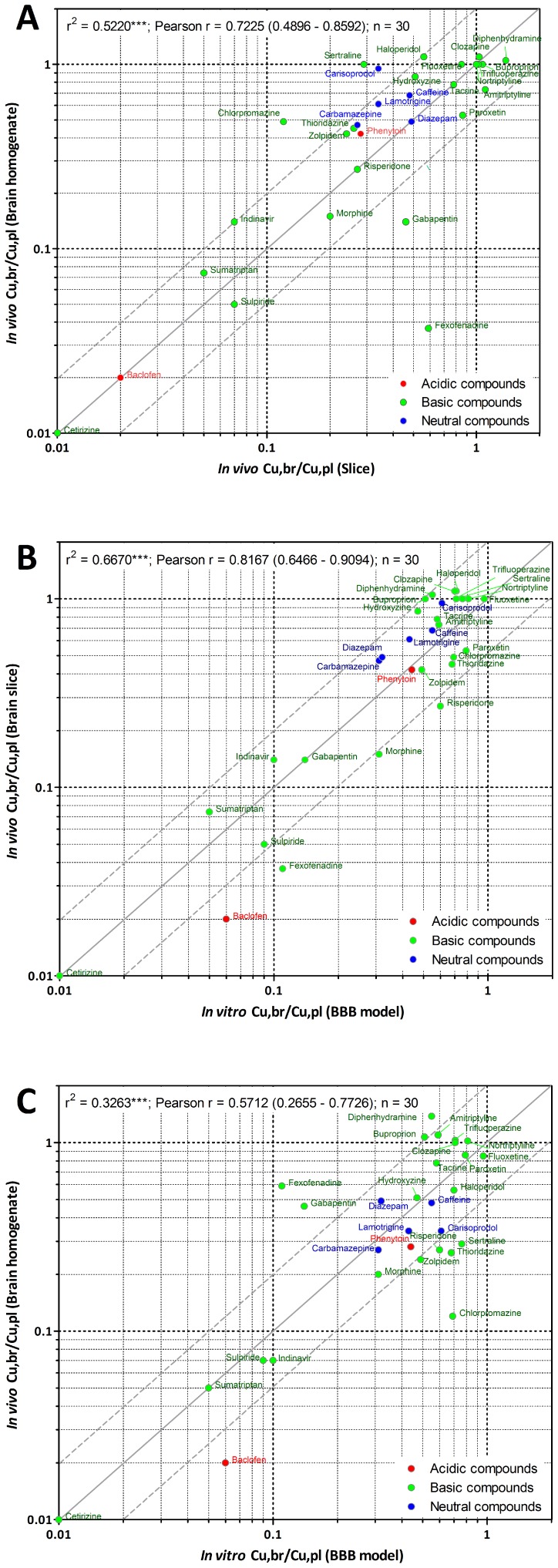
Relationship between *in vivo* Cu,br/Cu,pl obtained using fu,b from either brain slice or brain homogenate (A); Relationship between *in vitro* predicted steady-state Cu,br/Cu,pl and *in vivo* Cu,br/Cu,pl obtained using fu,b from either brain slice (B) or brain homogenate (C). The solid line represents perfect agreement. The dashed lines represent a 2-fold over- or underestimation compared with *in vivo* Cu,br/Cu,pl.

As can be seen in Fig. 3A, the correlation coefficients between the *in vivo* Cu,br/Cu,pl obtained using either fu,br from slice or from homogenate was rather weak r2 = 0,5220 but more than two-fold difference was obtained for only 5 of the 30 compounds assessed. These variations in *in vivo* Cu,br/Cu,pl determinations reflect the differences in the value of the fu,br depending on the methodology used.

The *in vitro* steady-state Cu,br/Cu,pl obtained for the 30 compounds were plotted against the corresponding *in vivo* Cu,br/Cu,pl obtained using fu,br from either brain slice (fig. 3B) or brain homogenate method (fig. 3C) and the correlation coefficients, r2, were 0.6670 and 0.3263 respectively.

The predicted *in vitro* steady-state Cu,br/Cu,pl were within two fold of the corresponding *in vivo* Cu,br/Cu,pl for 87% (i.e 26) of 30 compounds when compared to the *in vivo* data using brain slice (figure 3B) and for 73% (i.e 22) of 30 compounds when compared to the *in vivo* data obtained using brain homogenate (figure 3C). These predictions could be regarded as accurate given the precision of the *in vivo* Cu,br/Cu,pl determinations.

Since the brain slice method has been shown to generate more reliable fu,br data than the brain homogenate method based on a comparison with microdialysis results for 15 compounds [7], this method was routinely applied within AstraZeneca to determined fu,br of proprietary compounds within the discovery phase of CNS projects. To further assess the usefulness of the *in vitro* predicted Cu,br/Cu,pl, a comparison with the *in vivo* Cu,br/Cu,pl obtained using brain slice was undertaken for 62 proprietary compounds.

**Figure 4:**
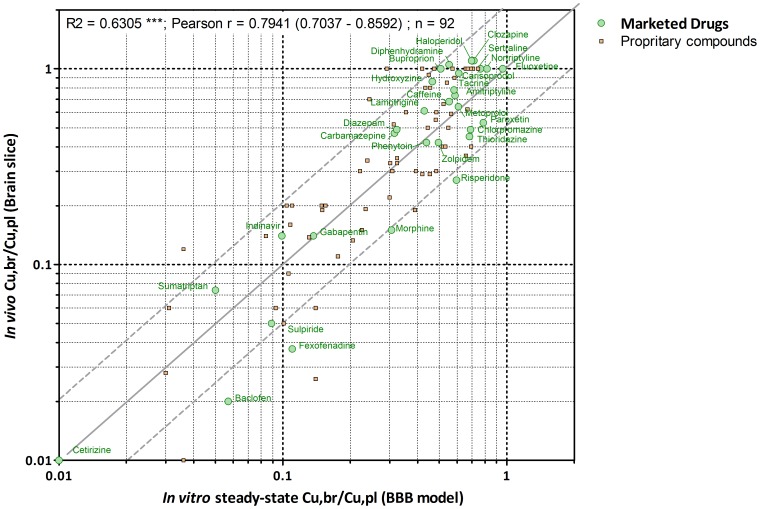
Relationship between *in vitro* predicted steady-state Cu,br/Cu,pl and *in vivo* Cu,br/Cu,pl obtained using fu,b from brain slice for 30 drugs and 62 proprietary compounds. The dashed lines represent a 2-fold over- or underestimation compared with *in vivo* Cu,br/Cu,pl. Individual values for drugs as well as proprietary compounds are defined in Table 2 and 3.

Overall, in a data set of 92 compounds (i.e 30 drugs and 62 proprietary compounds), comparing only steady state *in vivo*/*in vitro* Cubr/Cupl ratios, the correlation between *in vitro* and *in vivo* Cu,br/Cu,pl (using slice) was again rather high, r2 = 0.6305 (figure 4). Furthermore, 88% of the *in vitro* predictions were within two- fold of the corresponding *in vivo* ratios (using fu,br values from brain slices).

Compound screening in drug discovery frequently use *in vitro* experiments at the top of the screening cascade to measure efficacy. At this point, *in vitro* determined Cubr/Cupl ratios could be used together with *in vitro* efficacy data and predictions of free plasma concentrations to select molecules with the highest potential to elicit a pharmacological response when tested *in vivo*.

The results obtained for AZ13032000, a neuronal extracellular receptor antagonist found to have an IC50 of 55 nM (*in vitro*) were used to illustrate that *in vitro* determined Cubr/Cupl ratios could be used together with free plasma concentrations and *in vitro* efficacy data to predict whether resulting free brain levels are likely to elicit a pharmacological response.

This compound was found to have an *in vitro* steady-state Cu,br/Cu,pl ratio of 0.15 which could be multiplied by the free plasma concentration to yield estimated Cu,br.

**Figure 5:**
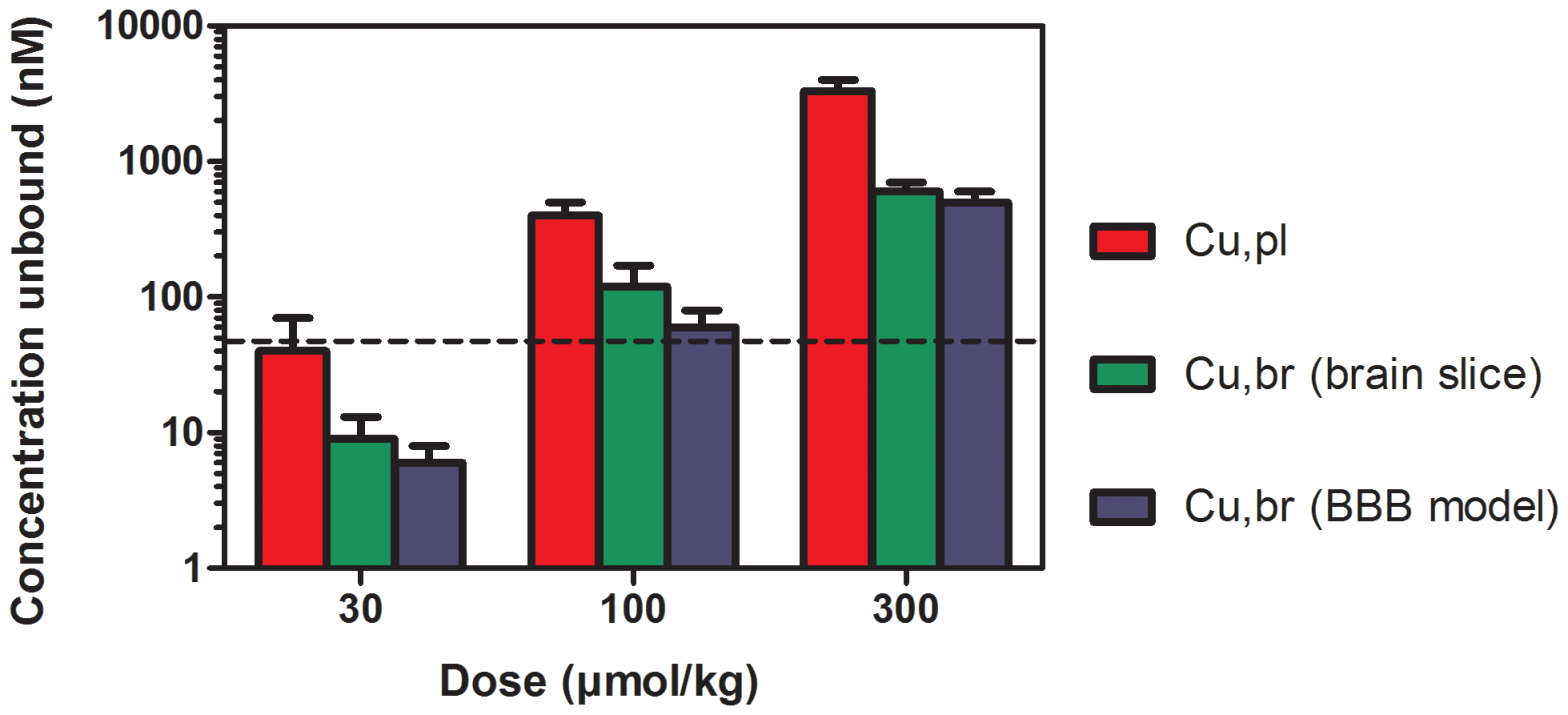
Comparisons between predicted free brain exposures (from Cu,br/Cu,pl *in vitro*) and *in vivo* exposures in relation to estimated IC50 in brain for AZ13032000 = 55 nM based on *in vitro* potency data.

As shown in the figure 5, three doses: 30, 100 and 300 µmol/kg of AZ13032000 were administered in rats and by multiplying the resulting in free plasma concentrations of 40, 400 and 3300 nM respectively by the *in vitro* steady-state Cu,br/Cu,pl ratio the Cu,br of 6, 60 and 495 nM were predicted for the lowest, medium and high dose respectively. When these brain levels were related to the IC50 value relevant pharmacological effects were anticipated at the medium and highest dose (i.e 100 and 300 µmol/kg). These results were confirmed in subsequent *in vivo* experiments, where the medium and high doses resulted in reductions in receptor activity around 60–70%. For the lowest dose, there was no significant reduction in activity compared to vehicle as anticipated by the predicted Cu,br of 6 nM. In addition, it can be seen that the predictions of free brain levels using the BBB model are in reasonable agreement with the free brain levels obtained by using *in vivo* total brain/plasma concentrations together with the unbound fraction determined by brain slices.

## Discussion

It has been recognized for some time that there is a clear need to improve the understanding of how drug exposures relate to target engagement and try to develop new approaches to predict whether a certain dose/plasma exposure is likely to achieve a desired pharmacological effect in the CNS. Ideally, *in vitro* potency data would translate to or predict *in vivo* potency, and often this is the case for a number of therapeutic targets. However, when there is no correlation between *in vivo* and *in vitro* potency measures, the validity of the *in vitro* assay, the *in vivo* model and target may be questioned. In view of this, it is of paramount importance to establish robust *in vitro*-*in vivo* relationships that aids target validation and boosts confidence in the *in vitro* and *in vivo* methodologies used in each drug development project. *In vitro*-*in vivo* relationships have maximum predictability when the relationship between pharmacological response and biophase concentration is known. Total plasma or free plasma concentrations are the most widely used surrogates for biophase concentrations although systemic concentrations may deviate significantly from the levels found in the biophase. Such differences in concentrations are frequently found for compounds that act on CNS targets. Due to the blood-brain barrier, CNS access of many compounds is restricted, which results in temporal dissociation between the biophase and systemic concentrations. The free drug hypothesis and the recent developments of techniques for easily assessing free brain concentrations, has led to significant improvements in terms of establishing such relationships between exposure and pharmacodynamic readouts.

The main objective of this study was to explore the possibility to generate Cu,br/Cu,pl ratios directly in a single *in vitro* model of the BBB, using a co-culture of brain capillary endothelial and glial cells in an attempt to mimic the *in vivo* situation and thereby greatly simplifying existing experimental procedures.

The *in vitro* non-steady state (i.e 1 h) and steady-state Cu,br/Cu,pl generated in a single experiment using the BBB model appears to correlate well with *in vivo* time-concentration profiles from microdialysis experiment. The difference between the *in vitro* Cu,br/Cu,pl ratios of clozapine at 1 h and at steady state seems to reflect the slow equilibration of clozapine between blood and brain. This may be an important quality of the BBB model, since lag phases such as this could have PK/PD implications.

The reasons for differences in the determinations Cu,br/Cu,pl with fu,brain using either brain slices or homogenates in this study are multiple, but probably includes factors such as: time to reach equilibrium (homogenate using dialysis over night and brain slices typically 5 h incubations), homogenization causing disruption or release of cell components which might cause differences compared to brain slices where cells are largely intact.

A stronger correlation was found between *in vitro* Cu,br/Cu,pl and *in vivo* Cu,br/Cu,pl from brain slice than with *in vivo* Cu,br/Cu,pl from brain homogenates. This could be at least partly explained by the preservation of cellular structures in the slice as well as in the *in vitro* BBB model that are absent in the homogenate, as it has been put forward that drug pH partioning, plays an important role for discrepancies between the two methods to determine fu,br [8]. Regardless of the factors causing these discrepancies, the results of the present study suggests that these kind of *in vitro* systems have the potential to predict the extent of free drug disposition in the brain in a single experiment and can be used to identify the compounds in the early drug discovery process that are most likely to show a desirable pharmacological response in the CNS.

Finally, given the growing interest in trying to exploit biotherapeutics for treatment of neurodegenerative disorders, it may be increasingly important to also be able to assess the free brain levels of these macromolecules in order to establish PK-PD relationships. However, to date, it has proven very challenging to assess the brain exposure of these macromolecules *in vivo* and microdialysis is rarely an alternative. At best, it is usually a very time consuming and labour intensive process to get reproducible *in vivo* results. However, the new *in vitro* BBB model opens up new possibilities for screening of biotherapeutics and could potentially, facilitate this process significantly.

Hopefully, this new approach can further contribute to advance PK/PD modelling as well as the process of providing high throughput platforms, which can drive the selection of, low- and high-molecular weight CNS therapeutics with the most favourable target engagement.

## Supporting Information

File S1 **Details of the calculation method used to predict the** ***in vivo*** **steady-state Cu,br/Cu,pl ratios from** ***in vitro*** **experimental data after one hour are given in the supplementary File S1.**
(DOC)
