# Supplementary material for: A Simple Method for Assessing Free Brain/Free Plasma Ratios Using an In Vitro Model of the Blood Brain Barrier
Source: PLoS One. 2013 Dec 3;8(12):e80634. doi: 10.1371/journal.pone.0080634 (PMC3849192; doi:10.1371/journal.pone.0080634)
Supplement: File S1 — Details of the calculation method used to predict the in vivo steady-state Cu,br/Cu,pl ratios from in vitro experimental data after one hour are given in the supplementary File S1. (DOC) [file pone.0080634.s001.doc]

**Supplementary Methods**

Data analysis

The in vivo steady-state Cu,br/Cu,pl were predicted from the in vitro experimental data collected after one hour according to the following equation:

Where:

Cdonor, t and Creceiver, t are the concentration of drug in the donor and receiver chambers at time t. Vreceiver,t and Vdonor,t are the volumes of each chamber at time t. Cdonor, t-1; Creceiver t-1 are respectively the drug concentrations in the donor and receiver chambers at previous time and Vdonor,t-1; Vreceiver,t-1 the volume of the donor and receiver chambers at this previous time.

No change in the volume of the donor or receiver compartments during the one hour transport experiment was observed in our experimental conditions.
